# Copper Oxide Chitosan Nanocomposite: Characterization and Application in Non-Enzymatic Hydrogen Peroxide Sensing

**DOI:** 10.3390/s17102198

**Published:** 2017-09-24

**Authors:** Antonella Arena, Graziella Scandurra, Carmine Ciofi

**Affiliations:** Department of Engineering, Messina University, Messina 98166, Italy; gscandurra@unime.it (G.S.); cciofi@unime.it (C.C.)

**Keywords:** electrochemical sensor, chitosan, modified electrodes

## Abstract

Electrochemical dissolution of metallic copper into slightly acidic aqueous solutions of chitosan yields a clear and stable dispersion of Copper Oxide nanoparticles into the organic polymer host. The electrochemically synthesized chitosan:CuOx nanocomposite is characterized by means of spectrophotometry, frequency domain electrical measurements and morphological analysis. Solid state electrochemical cells having pure chitosan as the electrolyte and using chitosan:CuOx as the electrode, are developed and characterized by means of electrical measurements performed in the ±1 V voltage window. The current-voltage loops of the cells, measured in deionized water, are found to reversibly change in response to hydrogen peroxide added to the water in 0.2 μM subsequent steps. Such changes, clearly distinguishable from changes recorded in response to other analytes, can be exploited in order to develop a hydrogen peroxide sensor able to work without the need for any supporting electrolyte.

## 1. Introduction

Hybrid materials, consisting of nanosized inorganic compounds dispersed into organic polymer hosts, have been the subject of renewed interest over the last few decades, owing to their facile preparation and to their potentiality in a variety of fields, going from fuel cells and supercapacitors [1,2], to photovoltaic energy generation [3,4], sensing [5,6], and catalysis [7,8]. Whenever the hybrid material has to be used in applications that involve interaction with living organisms, as is the case in the field of tissue engineering, in drugs delivery, and in food industry, the polymer host matrix is required to be biocompatible and to show no toxicity and allergenicity. All such requirements are fulfilled by chitosan [9,10,11]: an abundantly available biodegradable polymer, obtainable by partial deacetylation of chitin, a naturally occurring polymer found in crustacean shells, in fungal micelia and in other materials of biological origin [12]. Chitosan is a polysaccharide characterized by the presence of hydroxyl and amino functional groups in its chains. From the chemical point of view, it has the ability to interact with metal ions, organic halogen substances, and biological molecules, through a variety of mechanisms including chelation, electrostatic attraction, and ion exchange. For these reasons, chitosan has been successfully used in environmental applications, such as the removal of contaminants from wastewater [13]. In addition, chitosan is a substrate commonly used for enzymes immobilization [14]. Due to these properties, and to its ability to form stable films, insoluble in water, with good adhesion and high mechanical strength, chitosan is also an ideal candidate in sensing and biosensing applications. There are, in fact, several examples of gas [15,16,17,18] and humidity [19] sensors based on thin films of chitosan, and a wide variety of enzymatic [20,21,22] and enzyme-free [23,24,25] electrochemical sensors based on chitosan-transition metal complexes, and on chitosan dispersions of nanoparticles. As far as electrochemical sensing behavior is concerned, chitosan has been applied in the detection of hydrogen peroxide both in the enzymatic [26,27] and in the non-enzymatic versions [28,29,30]. In this paper, we demonstrate that the presence of hydrogen peroxide in water, can be successfully detected at a micromolar level by means of a solid state cell in which chitosan is used both as the polyelectrolyte that provides the electrical connection between the electrodes, and as the host polymer of the electrochemically active nanocomposite used as the electrode. Compared to other hydrogen peroxide sensors based on chitosan, the cell described here takes advantage of chitosan being used as the ion conducting bridge between the electrodes, and therefore it is able to work without the need for any kind of supporting electrolyte [31,32].

## 2. Materials and Methods

### 2.1. Preparation of the Chitosan:CuOx Sensor

**Figure 1:**
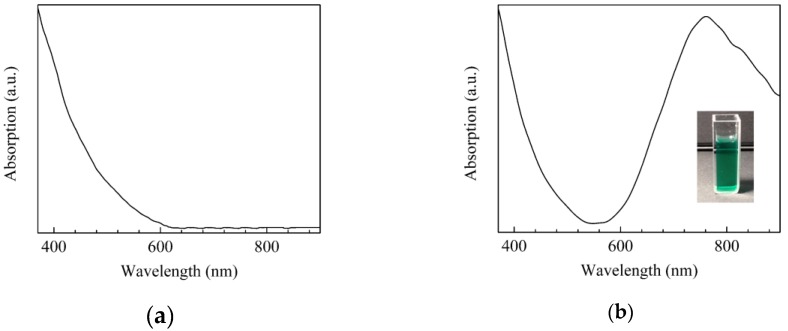

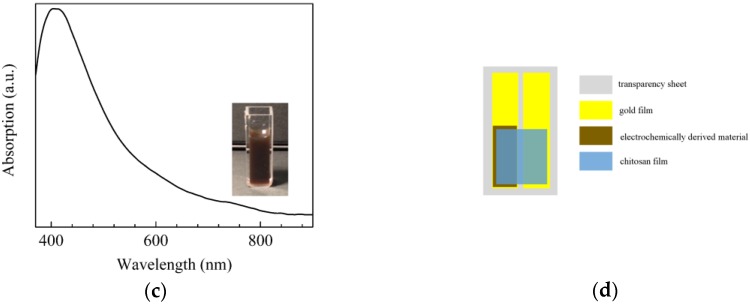
Absorption spectrum of the chitosan aqueous solution (**a**); photo and absorption spectrum of the green (**b**), and of the brown (**c**) materials electrochemically derived from copper wire electrodes immersed in chitosan solutions; (**d**) schematic view of the solid state cells based on chitosan.

Chitosan (medium molecular weight, purchased from Aldrich, St. Louis, MO, US), is soluble in acidic aqueous environment, where, at pH lower than 6.5, the amino groups of the polymer are protonated to NH_3_^+^, and the polymer behaves as a cationic polyelectrolyte. Electrochemical synthesis was performed by placing two copper wire electrodes (Aldrich) inside a beaker containing 100 mg chitosan solubilized in 150 mL water and acetic acid (pH ranging from 5 to 6), and by applying a constant electric field between the two electrodes while the solution was agitated through a magnetic stirrer, in order to prevent the chitosan molecules from sticking on the surface of the copper cathode. Depending on the acetic acid concentration, two different kinds of electrochemical processes, involving the release of copper from the electrodes, were observed. At pH lower than 5.5, a pale blue hydrogel formed, similar to that obtained by Geng et al. [33], and identified by them as a copper/chitosan complex. As the electrochemical process proceeded, the pale blue solution slowly darkened, reaching the green after a few hours (Figure 1b). At pH higher than 5.5, under an applied electric field of about 5 V/cm, the copper anode started to quite rapidly consume, while the initially uncolored chitosan solution began to darken and became unclear, finally assuming a dark brown color, after a few minutes (Figure 1c).

Both the electrochemically obtained materials were used to develop electrochemical cells to be used to detect the presence of hydrogen peroxide in water. For each material, the cells were obtained as follows. We started from parallel rectangular shaped gold electrodes, spaced by half a millimeter, previously evaporated in vacuum onto copier grade transparency sheets (Tartan); a drop of the solution (either the “green” or the “brown” one) was deposited onto the top of one of the gold electrodes; after all the residual solvent evaporated, chitosan was deposited in drops in such a way as to form an ion conducting bridge between the exposed gold electrode and the one covered with the chitosan based material. A schematic view of the cell is shown in Figure 1d.

### 2.2. Characterization of Chitosan:CuOx Sensor

The chitosan based materials developed as described in the previous section, were characterized by means of spectrophotometric measurements performed in the visible range by using an HR4000 microspectrophotometer (Ocean Optics, Largo, FL, US). Atomic Force Microscopy (AFM) analysis, carried out by means of a Nanosurf FlexAFM equipped with a C3000 controller (Nanosurf AG, Liestal, Switzerland), was performed to investigate the morphology of the chitosan based films, deposited from the solutions onto silicon substrates. The electrical properties of the developed materials were investigated by using impedance measurements, performed in air by means of an Agilent 4284A LCR meter (Agilent Technologies, Santa Clara, CA, US), in the frequency range between 20 Hz and 1 MHz, with a 100 mV amplitude. The impedance measurements were performed on samples obtained by drop-depositing the materials from the liquid phase, onto the insulating gap between parallel gold electrodes spaced by a few hundred microns, thermally evaporated on copier grade sheets.

### 2.3. Chitosan:CuOx Sensing Tests

A 2400 source meter (Keithley, Cleveland, OH, US) was used to measure the current of the chitosan based solid state cells, in response to zero average triangular voltage inputs. Measurements were carried out at different voltage time rates, over the ±1 V voltage windows. Sensing tests were performed in deionized water, into which hydrogen peroxide, was injected by using a micro syringe, increasing its concentration in steps of 0.2 × 10^−6^ M. Ammonia and acetic acid where used for testing the behavior of the sensor in the presence of interferent analytes.

## 3. Results and Discussion

The electrochemically derived materials based on chitosan and copper were developed starting from chitosan acidic aqueous solutions transparent in the visible spectral range, as it is shown in the spectrum of Figure 1a. Depending on the pH of the chitosan acidic aqueous solution in which the electrochemical synthesis takes place, two kinds of products are obtained: the clear green solution and the dark brown suspension shown in the photos of Figure 1b,c. The nature of the two electrochemically derived products can be understood on the basis of the ability of chitosan to coordinate transition metal ions, forming metal complexes, on the condition that the pH of the solution in which the chemical reaction takes place meets certain requirements. It has been demonstrated that starting from copper salts dissolved in chitosan’s solutions, in slightly acidic condition [34], the polymeric amine groups coordinate bivalent copper ions, forming metal complexes. On the contrary, in basic condition, these complexes do not form, and copper hydroxides precipitate. The chitosan–copper complexes exist in two configurations differing from each other by the number of amino groups that coordinate the metallic ions. Both these metallic complexes have optical absorption spectra characterized by the presence of a broad band, ranging from the red to the near infrared spectral interval, ascribable to d–d electron transitions of the Cu^2+^ ions. Such a band is pH sensitive, as it is found to shift from the near infrared to the visible region, as the pH of the solution increases. Based on these considerations, the intense and broad absorption band, centered at about 760 nm in the optical absorption spectrum shown of Figure 1b, can be attributed to the copper ions, confirming that the green solution is a copper–chitosan complex. On the other side, the band at about 760 nm is not observed in the spectrum of the brown material, shown in Figure 1c. According to Omar et al. [35], the near infrared band ascribable to the complexation of copper with chitosan, disappears when a reducing agent is used to convert the chitosan-copper complex into chitosan, copper, and copper oxide. This consideration, and the presence of a single absorption band positioned at about 445 nm, similar to that found by Basumallick et al. [36] and ascribed to chitosan coated copper oxides, suggest that the electrochemically synthesized brown material can be identified as chitosan dispersion of copper oxides particles. In particular, the electrochemically derived brown suspension, which from now on will be referred to as chitosan:CuOx, is likely to be a chitosan dispersion of the red cuprous oxide (Cu_2_O) and the black coupric oxide (CuO), mixed with a predominance of the former one, as the brownish color of the material suggests.

**Figure 2:**
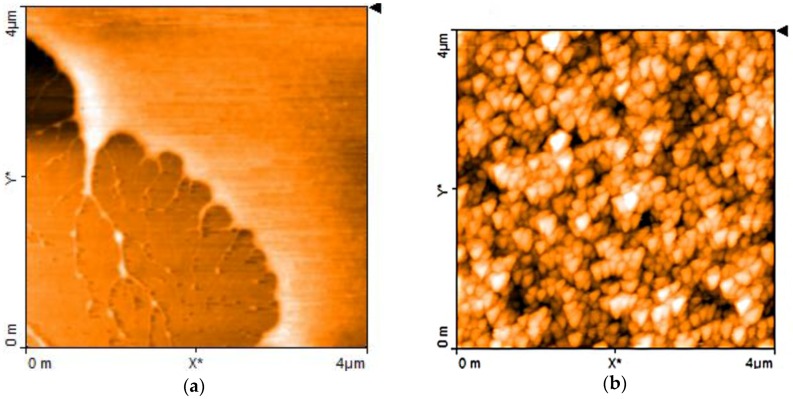
Atomic Force Microscopy (AFM) image close to the border of a thin film of chitosan-copper complex deposited onto a silicon substrate (visible in the bottom left portion) (**a**); AFM image of the chitosan:CuOx film(**b**).

The results of AFM morphological investigations confirm the hypothesis on the nature of the electrochemically derived materials. The films deposited from the copper/chitosan complex solutions are quite smooth, as is evidenced in the upper-rightmost portion of Figure 2a. On the contrary, films obtained from the brown liquid reveal the presence of regularly shaped particles, having average size smaller than 200 nm, as is evidenced in the AFM micrographs of Figure 2b.

**Figure 3:**
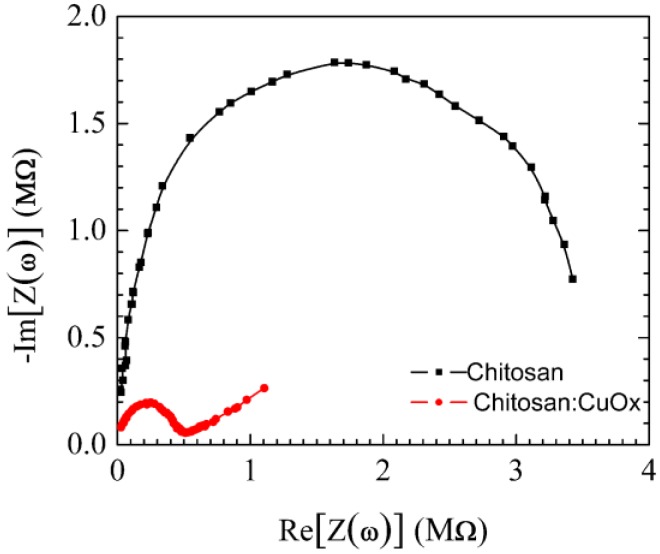
Opposite of the imaginary part of the impedance, plotted against the real part of the impedance, of chitosan and of chitosan:CuOx.

From the electrical point of view, depending on its average molecular weight, on its crystallinity, and on its degree of acetilation, chitosan in the hydrated form behaves as an ionic conductor. Responsible for charge transport are mobile hydroxide ions that, in the presence of water, originate from the partial protonation of the amino groups bounded to the polymeric chain [36]. Figure 3 shows the Cole plot of chitosan and of chitosan:CuOx.

The opposite of the imaginary part of the impedance of chitosan, plotted versus the real one, forms a wide semicircle, as happens in the case of samples having a parallel combination of resistance, capacitance and Warburg elements as electrical equivalent. Compared to that of pure chitosan, the chitosan:CuOx Cole plot has a smaller semicircle, and therefore it is expected to have improved charge transport properties.

**Figure 4:**
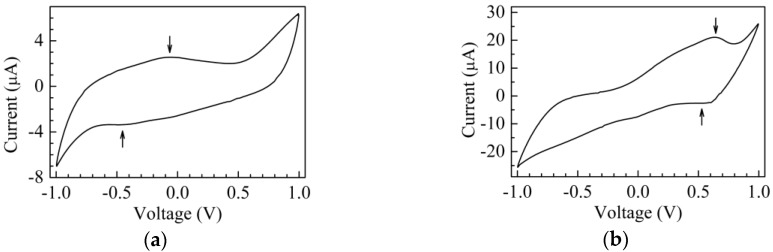
(**a**) Current-voltage loop of a cell obtained using the electrochemically derived copper-chitosan complex as the electrode; current-voltage plot of the Au/chitosan:CuOx/chitosan/Au cell (**b**).

Figure 4a,b shows respectively the current–voltage plot of a couple of cells developed as sketched in Figure 1c, one having the gold electrode coated with the copper/chitosan complex and the other having a chitosan:CuOx coated gold electrode.

**Figure 5:**
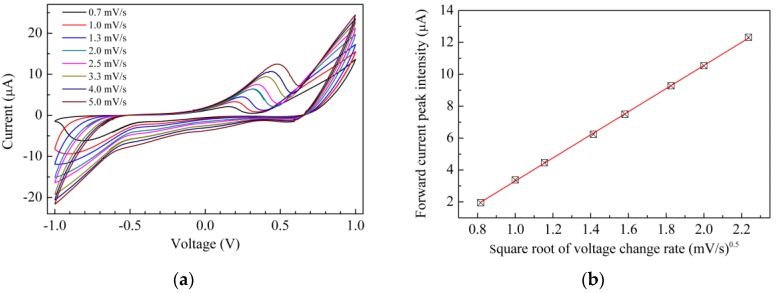
(**a**) Current-voltage loop of the Au/chitosan:CuOx/chitosan/Au cell, measured at different scan speeds; (**b**) Intensity of the forward current peak of Figure 5a, plotted against the square root of the scan speed.

The curves are obtained in deionized water, by measuring the current in response to zero average triangular voltage inputs with 40 s period (±50 mV/s voltage rate of change with time). After a transient phase in which the current initially grows with the increasing voltage, the current starts to cycle, forming the loops shown in Figure 4. Current loops form whenever the current is affected not only by the voltage, but also by the time rate of the voltage change: such a situation is commonly observed when dealing with capacitive-resistive systems. This may be the case in the chitosan based cells, where double layer capacitance forms at the interface between the electrode and the conducting polyelectrolyte. In addition, whenever the electrode contains electroactive species undergoing redox processes over the swept voltage range, characteristic current peaks usually arise. Observing the current–voltage plot of Figure 4a, a weak forward peak (marked by an arrow) can be noticed at about −45 mV and a weaker reverse peak is observed at about −450 mV: such a couple of peaks may be consistent with the hypothesis of redox processes involving the Cu^2+^ ions. Compared to that of Figure 4a, the I–V loop measured using the chitosan:CuOx modified electrode, shown in Figure 4b, has a five times larger current intensity, and exhibits a forward current peak positioned at 600 mV, approximately. A shoulder, marked by the arrow in Figure 4b, indicates that a reverse current peak is likely positioned between 500 mV and 600 mV. The separation of the forward and reverse current peaks, and their intensity ratio (considerably below unity), indicate that the redox processes responsible for the peaks are irreversible. To investigate the nature of such processes, current–voltage measurements have been performed using 1 V amplitude triangular voltage inputs, with a different rate of change of voltage with time. Figure 5a collects the results. Starting from the inner loop measured at the lowest time rate of change (±0.6 mV/s), and proceeding outward (i.e., as the voltage time rate of change increases), the loops enlarge and the current peaks increase their intensity and move towards a larger voltage. Figure 5b shows the intensity of the forward current peak of Figure 5a, plotted against the root square of the modulus of the voltage rate of change with time. The experimental data are distributed along a straight line, as is evidenced by the best fitting curve, indicating that a diffusion limited electrochemical process is responsible for the forward current peak. According to the results shown in Figure 4 and Figure 5, both the chitosan complex and the chitosan:CuOx dispersions seem to be electroactive in the voltage window between ±1 V. We believe it could be of interest to investigate the possibility to exploit the solid state cells based on such materials as electrochemical sensing systems.

**Figure 6:**
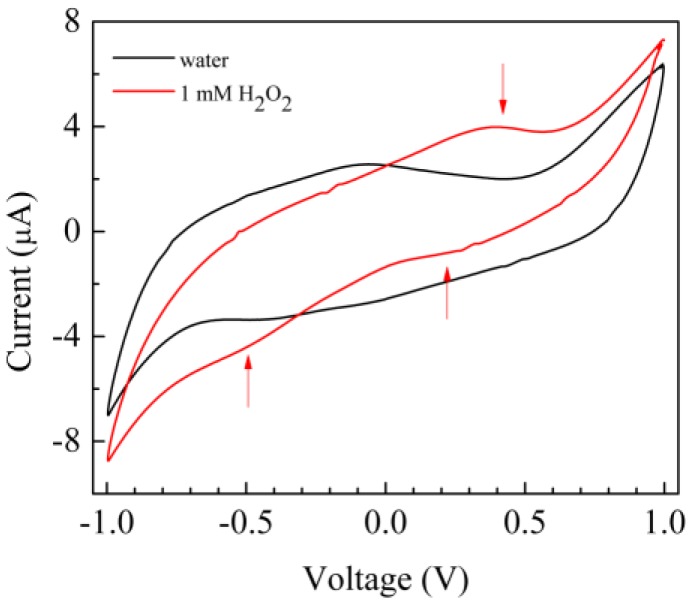
Current-voltage loop of the Au/chitosan–Cu-complex/chitosan/Au cell, measured in pure water, and in the presence of 0.5 mM hydrogen peroxide.

Recently, Geng et al. [33] have shown that the presence of hydrogen peroxide in water can be detected by three electrode measurements in 0.1 M phosphate buffer, by using a copper complex based on chitosan applied on a titanium plate as a sensitive electrode. Figure 6 shows the results of sensing tests performed by us, measuring the current-voltage loop of a typical Au/chitosan–copper complex/chitosan/Au cell into deionized water, with and without 1 mM of hydrogen peroxide. It can be noticed that in response to H_2_O_2_, the loop modifies: in particular, a forward current peak (marked by an arrow in Figure 6), arises at about 500 mV. In addition, it is found that the current–voltage loop turns back to its original shape (black line in Figure 6), when the solution containing hydrogen peroxide is replaced with deionized water.

**Figure 7:**
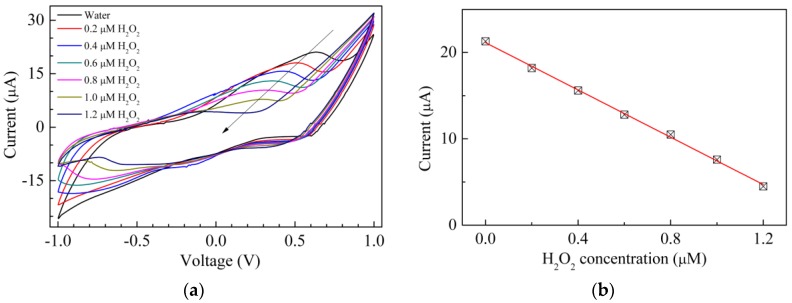
(**a**) Current-voltage loop of the Au/chitosan:CuOx/chitosan/Au cell, measured in water, and in the presence of hydrogen peroxide at different concentrations; (**b**) Intensity of the forward current peak intensities of Figure 7a, plotted against the H_2_O_2_ concentration.

It can be inferred that the solid state cell schematized in Figure 1d, having the electrochemically derived chitosan-copper complex coated on the top of one of the gold electrodes, is able to detect H_2_O_2_ in water without the need of any supporting electrolyte, when the analyte’s concentration is in the range of 10^−3^ M. Measurements performed exposing the cells to different concentrations of the analyte show that the solid state cells based on the chitosan-copper complex reversibly respond in a reproducible way to hydrogen peroxide in the millimolar range, with a limit of detection of 0.2 mM. Sensing tests performed on solid state cells having chitosan:CuOx as the active electrode, reveal that the copper oxide dispersions have higher sensitivity towards H_2_O_2_ compared to the copper–chitosan complex. The set of measurements of Figure 7a are performed by exposing a solid state cell, having chitosan:CuOx as the active electrode, to hydrogen peroxide at a concentration that increases in steps of 0.2 μM. Unexpectedly, however, starting from the current-voltage loop measured in pure water, the reverse current progressively decreases and the original forward current peak rapidly weakens as the hydrogen peroxide concentration increases. In particular, the intensity of the forward current peaks, plotted against the hydrogen peroxide concentration, as shown in Figure 7b, decreases in a linear way with a slope of about 1 μA per 0.1 μM of H_2_O_2_. On the contrary, according to the scientific literature, electrochemical measurements carried out in the presence of supporting electrolytes show that sensors based on graphite and glassy carbon electrodes modified with copper oxide nanoparticles [37,38,39], and with nanosized copper oxide dispersed into mixtures of multiwalled carbon nanotubes and chitosan [40], do respond to increasing amounts of hydrogen peroxide with an increasing current.

The experimentally observed behavior of Figure 7 suggests that somehow the electrochemically derived chitosan:CuOx, applied to the top of the gold electrode, as schematized in Figure 1d, does interact with H_2_O_2_, but the way in which this interaction manifests itself is a sort of reversible quenching of the electroactivity of chitosan:CuOx.

**Figure 8:**
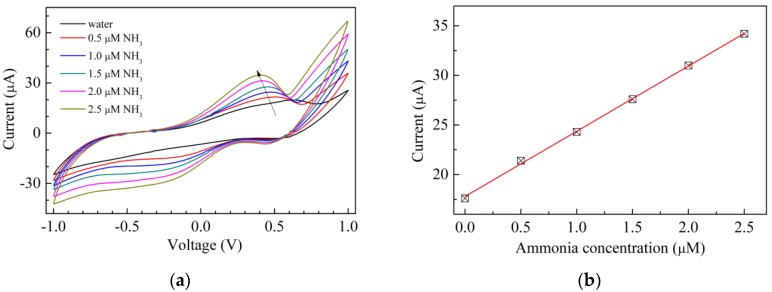
(**a**) Current-voltage loop of the Au/chitosan:CuOx/chitosan/Au cell, measured in pure water, and in the presence of ammonia at different concentrations; (**b**) Intensity of the forward current peak intensities of Figure (a), plotted against the NH_3_ concentration.

**Figure 9:**
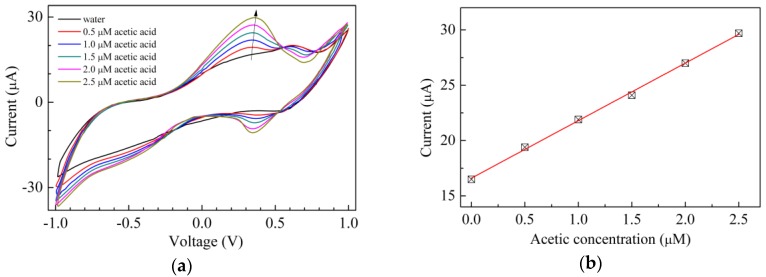
(**a**) Current-voltage loop of the Au/chitosan:CuOx/chitosan/Au cell, measured in pure water, and in the presence of acetic acid at different concentrations; (**b**) Intensity of the forward current peak intensities of Figure 9a, plotted against the acetic acid concentration.

Aimed at evaluating the behavior of the Au/chitosan:CuOx/chitosan/Au cells towards analytes other than H_2_O_2_, sensing tests have been carried out in the presence of a base and of a weak acid, namely ammonia and acetic acid. The results are shown in Figure 8 and Figure 9 respectively. It can be noticed that in both cases the forward current peak found at about 600 mV in the current–voltage loop of Au/chitosan:CuOx/chitosan/Au in water disappears and new current peaks, the positions of which depend on the analyte, arise. In particular, Figure 8a shows that when exposed to ammonia in the micromolar range, the current–voltage loops of the Au/chitosan:CuOx/chitosan/Au cells enlarge, and a forward current peak arises, the intensity of which is linearly related to the ammonia concentration, as the results of Figure 8b suggest. In the presence of acetic acid, as is illustrated in Figure 9a, a couple of forward and reverse current peaks arise, and again, according to the results shown in Figure 9b, the intensity of the forward current peak is found to be linearly related to the acetic acid concentration. In both the examined cases, the observed modification is reversible, as after a transient time the current-voltage loops turn back to their original shape and intensity by immersing the cells in deionized water. These latter results, being the investigated analytes a base and a weak acid, could be explained in terms of the pH sensitivity of nanosized copper oxide [41], which may persist when the material is dispersed in the chitosan host and used in the solid state cell configuration of Figure 1d.

The fact that in the case of H_2_O_2_ the amplitude of the peaks is decreasing for increasing concentration while the opposite occurs in the case of a weak acid and a base, can be used to discriminate the presence of hydrogen peroxide with respect to other species. With regard to the feasibility of employing the investigated structure for the realization of an H_2_O_2_ sensor, we must observe that, clearly, the fact that the response decreases with increasing concentration sets an upper limit to the maximum detectable concentration and, hence, to the dynamic range. This possible disadvantage is partially compensated by the high sensitivity that is observed at lower concentrations, as summarized in Table 1.

## 4. Conclusions

It is demonstrated that solid state cells with simple design, having electrochemically derived materials based on copper and chitosan applied to the top of one gold electrode, can be successfully used to detect the presence of hydrogen peroxide in water, at concentrations below 1 μM, without the need of any supporting electrolyte. While characterized by high sensitivity, the cells using chitosan:CuOx dispersions are found to respond to the presence of H_2_O_2_ with a decreasing current, unlike other conventional electrochemical sensors based on nanosized copper oxides. The difference between the two kinds of behavior can be likely ascribed to the fact that copper oxide still mantaints its electroactivity towards H_2_O_2_ but the way in which the material responds to the analyte is affected by the absence of a supporting electrolyte.

## Figures and Tables

**Table 1:** Sensitivity of the Au/chitosan:CuOx/chitosan/Au cell towards H_2_O_2_, compared to that of other electrochemical sensors.

Sensor	Sensitivity (μA·mM^−1^ cm^−2^)	Ref.
Au/chitosan:CuOx/chitosan/Au	10,000	This work
Glassy carbon electrode modified with copper nanoparticles decorated silver nanoleaves	6190	[42]
Glassy carbon electrode modified with FeS nanosheets	36.4	[43]
Carbon paste electrode modified Ni-Al/layered double hydroxide/Ag nanoparticles	1.836	[44]
Glassy carbon electrode modified with Ag@C core-shell nanomaterials	22.94	[45]
Graphene oxide electrode modified with Binary Mn-Co Oxides	53.65	[46]
